# Correlation of inflammatory cytokines and uric acid levels in coronary artery disease with and without type 2 diabetes mellitus

**DOI:** 10.6026/97320630018675

**Published:** 2022-08-31

**Authors:** D Selvakumar, CK Vijayasamundeeswari, Sandip Sendhav, Gnanadesigan Ekambaram

**Affiliations:** 1; 2; 3

**Keywords:** Coronary artery disease, hs-CRP, Interleukin-6, Type 2 Diabetes Mellitus, Hypertension, Uric acid

## Abstract

Serum levels of inflammatory cytokines such as IL-6, high-sensitivity C-reactive protein as well as uric acids have been considered as predictors of severe outcomes in patients with coronary artery disease (CAD) associated with hypertension (HTN) and Type 2 Diabetes Mellitus (T2DM). Anthropometric parameters were recorded and measured the levels of major risk factors in 80 patients with hypertension, coronary artery disease with and without Type 2 diabetes mellitus and 40 healthy controls were included. Comparison were performed by classifying the study subjects into 3 groups namely- Group I: Controls (n=40); Group II: HTN, CAD without T2DM(n=40); and Group III: HTN,CAD with T2DM(n=40).The values of BMI, weight, SBP, DBP were greater in patients with T2DM CAD group when compared to without diabetes group. Data shows that there is a positive correlation between IL-6, hs-CRP and uric acid concentrations and are statistically significant. The evaluated high levels of inflammatory cytokines and uric acids in hypertensive CAD patients with diabetes might be helpful in the diagnosis of patients at greater risk.

## Background:

Coronary artery disease is the most frequent acute and chronic condition in the industrialised world, and it is the main cause of mortality and morbidity. The cardiovascular epidemic is a global phenomenon that accounts for about half of all deaths in developed countries. There had been 2.03 million fatalities in India in the year 2010 owing to CAD, with the prevalence of CAD being 2-3 times greater in urban than rural populations, with 96.7 percent per 1000 people in urban and 27.1 percent per 1000 population in rural [1]. Atherosclerosis, the underlying cause of most CHD, is a slow and quiet process that begins early in childhood and continues for decades. Inflammation has a key role in the development of atherosclerosis. Individuals at risk for atherosclerotic events have higher levels of many inflammatory markers in their blood. Interleukin-6 (IL-6) is a multifunctional cytokine that modulates immunological response, acute phase reactions, and hematopoesis. It plays a crucial function in the defence mechanisms of the host, and it's one of a number of pro-inflammatory cytokines have been linked to the development of insulin resistance [2]. Various studies have identified connections between IL-6 levels and CAD severity and have shown that patients with pre-existing CAD had increased levels of IL-6 compared to patients without CAD, coronary events mortality and progression to heart failure [3, 4]. Higher serum levels of proinflammatory cytokines including IL-6 have been linked to increased blood pressure and organ damage in hypertensive patients. IL-6 is a key regulator of the hepatic synthesis of acute phase proteins including C-reactive protein (CRP) which has been linked to hypertension and cardiovascular disease [5]. Obesity and insulin resistance have long been recognised as essential and primary causes of major health problems such diabetes, dyslipidemia, hypertension, and cardiovascular disease. Recent research has found that CRP levels were associated to BMI, insulin sensitivity, insulin and proinsulin fasting plasma levels. Hs-CRP boosts the proinflammatory effects of numerous mediators, promoting atherosclerosis progression [6]. The American Heart Association developed a scientific statement that recommends hs CRP as a sensitive assay for the prediction of vascular disease, compared to traditional assays for circulating C-reactive protein levels, following a systematic review of the association between inflammatory markers and coronary heart diseases [7]. Uric acid is a by-product of purine metabolism that is processed by the hepatic enzyme xanthine oxidase and its activity gets increased during ischemia and intensifies following reperfusion. The presence of a baseline increase in serum uric acid levels implies the occurrence of coronary heart disease. Increase in serum uric acid concentration of 50 mm/L is linked to a 14 percent increase in cardiovascular mortality. High concentration of uric acid is a strong indicator of prognosis for moderate to severe heart failure and cardiovascular disease [8]. Diabetes mellitus raises the risk of (CAD) by causing endothelial dysfunction and dyslipidemia, both of which are early stages in the atherogenic process. DM is one of the highest CAD risk factors due to these pathways, with a very high 10-year risk of CV events. Lower levels of high-density lipoprotein (HDL-C), low-density lipoprotein cholesterol (LDL-C), elevated triglyceride levels are linked to a higher BMI, thereby increasing the risk of CAD [9,10]. Previous studies inconsistency and individually reported risk factors for CAD particularly in hypertension patients with and without T2DM. Therefore, it is of interest in analysing the relationship between inflammatory cytokines (IL-6 and hs-CRP) and uric acid levels in CAD patients with and without T2DM.

## Materials and Methods:

The study comprised of 120 subjects involving both males and females. The subjects were classified into three groups: Group 1: Controls (n=40), Group II: HTN, CAD without T2DM (n=40), and Group: III HTN, CAD with T2DM (n=40). The baseline characteristics such as age, gender, height, weight, BMI, waist-hip ratio, alcohol consumption, SBP, DBP, and smoking were collected. The inclusion criteria for selecting the subjects for the study were the age of the subjects were preferably between 30 and 70 years, Both the groups of patients with CAD proven by Angiogram. The exclusion criteria were patients who had history of autoimmune disease, liver disease, kidney diseases, other type of diabetes mellitus, Thyroid diseases, congenital heart diseases, other types of heart diseases, and also persons whoever were not willing to participate in the study were excluded. The study was conducted in PES medical college super specialty hospital, Kuppam, Andhra Pradesh. The study participants were recruited after approved by Institutional Ethics committee IEC Ref no: PESIMSR/IHEC/52/2017-18), and an informed consent form.

## Sample collection:

10ml of and 3ml of postprandial venous blood samples were collected from the cubital fossa from all the participants after 8 to 10 hours of fasting. Plasma and serum were centrifuged at 3000RPM for 10 minutes and separated samples into properly labelled aliquots, stored at -80oC until biochemical analysis was done. Plasma samples used for fasting, postprandial blood sugars and glycated haemoglobin, serum used for lipid profile, uric acid were analysed by laboratory standard methods (Vitrous-250 fully automatic analyzer). hs-CRP was analysed by turbidometric method and IL-6 were determined by enzyme linked immunosorbent assay (ELISA, EuroImmune Autoanalyzer).

## Statistical analysis:

The statistical analyses were carried out by using the software Statistical Package for the Social Sciences (SPSS) Version 20.0. The values were expressed as mean ± standard deviation, P < 0.05 was considered statistically significant. Comparison of study subjects was performed by one-way ANOVA and comparison between the three groups was done by post hoc analysis. Correlation of BMI, Uric acid, hs-CRP and IL-6 with other parameters of the study was done by Pearson’s correlation. The predictive values of clinical and descriptive parameters were evaluated by constructing (receiver operating characteristic (ROC) curve analysis and the area under the curve (AUC) was calculated.

## Results:

Table 1(see PDF) illustrated that There was a statistically highly significant the mean age of controls (47.27 ± 5.46) whereas it was (51.05 ± 6.63 , 55.57 ± 8.13) for hypertension CAD with and without type 2 diabetes mellitus patients respectively (P = 0.0001**). In case of BMI were highly significant different between hypertension CAD with and without type 2 diabetes mellitus patients (33.35 ± 4.01, 30.75±3.10) When compared to healthy controls (21.15± 1.85, P=0.0001**). Additionally we observed there was statistically significant of systolic blood pressure (172.22±6.66, 151.22±2.17) and diastolic blood pressure (101.02 ±6.69, 92.42±1.79) in between hypertension CAD with and without type 2 diabetes mellitus patients when compared to healthy controls ( 116.10 ±3.01 and 74.30±3.79, P=0.0001**). The major biochemical and clinical factors associated with CAD diseases were analysed and the results are tabulated in Table 2(see PDF). There were significantly elevated levels of FBS, PPBS, HbA1c, total cholesterol, LDL, uric acid, hs-CRP and IL-6 in group 3 and group 2 when compared to group 1, respectively P value is 0.0001**. This inferred that diabetes and hypertension patients were more prone for Coronary artery disease. To further investigation TGL and VLDL concentrations significantly increased in group 2 subjects when compared to group 3 and group 1, The P values were found to be highly statistically significant (P= 0.0001**). We further evaluated the influence factors of descriptive, biochemical and clinical parameters compared by Post hoc analysis is represented in Table 3. As described FBS, TC, TGL, HDL, VLDL,LDL, hs-CRP and interleukin-6 were strongly significant, there was moderate significants of BMI, HbA1c and no significant difference of age, PPBS and uric acid in group 1 vs group 2. When compared with group 1 vs group 3 shown highly significant of age, FBS, PPBS, HbA1c, TC, TGL, HDL, VLDL, LDL, uric acid, hs-CRP, IL-6 and also we observed that BMI were shown low significant . Additionally we compared group 2 vs group 3 participants shown strongly significants of FBS, PPBS, HbA1c, TC, TGL VLDL, LDL, uric acid, hs-CRP, IL-6, there was a significant of BMI and also no significant difference of age, HDL. To further investigate the relationships among BMI, Uric acid, hs-CRP, interleukin-6, and other indexes were analyzed, and the results were shown in Table 4(see PDF). As described, BMI, uric acid, hs-CRP and interleukin-6 were found to be strongly correlated to age, SBP, DBP, FBS, PPBS, HbA1c, TC, TGL, LDL, respectively (P value 0.0001**), there were negative correlation between HDL and BMI ,hs-CRP, interleukin-6 (r = - 0.792,- 0.831 and – 0.656, P= 0.0001**). Whereas there is no correlation was observed between uric acid and TGL (r= 0.135, P=0.142). The diagnostic utility of the uric acid, interleukin-6 and hs-CRP for the detection of early coronary artery disease was compared and results were tabulated in Table 5(see PDF). Among these hs-CRP, showed a strongly significant area under curve (AUC =1.000, P=0.0001**), there were interleukin -6 has moderate significant (AUC = 0.997, P=0.0001**). Additionally the uric acid shown statistically significant AUC (0.687), respectively P value is < 0.004*. The present study showed that IL-6 have a strong positive correlation with BMI, LDL, uric acid and hs-CRP and therefore increase in serum IL-6 cytokine level led to rise in the levels of other parameters under consideration. The results are shown in Figure 3(see PDF).

## Discussion:

The current study reveals that circulating plasma levels of IL-6 and CRP and uric acid levels in the studied subjects were associated with a number of clinical cardiovascular risk factors such as age, BMI, SDP, DBP, HbA1c, TC, FBS, PPBS and LDL. Patients with type-2 diabetes have a twofold increased risk of stroke and all indications of CHD, myocardial infarction (MI), sudden death, and angina pectoris than non-diabetic individuals [11]. Insulin resistance causes the breakdown of stored triglycerides in fat cells, resulting in an increase in free fatty acids in the blood plasma and connected to well-known atherosclerosis risk factors like hypertension, hyperlipidemia, and obesity, all of which hasten the onset and progression of atherosclerosis [12]. Previous studies investigated the impact of circulating interleukin-6levels and CRP, IR,SBP,DBP and also they reported inflammation is directly associated to insulin resistance and macro-angiopathy in type-2 DM, and hs-CRP can be a valuable marker for evaluating pathophysiology in type 2 DM or vascular disease [13]. Recent study has found that the diabetic patients have increased blood levels of IL-6, which is known to increase the inflammation and development of vascular disease and atherosclerosis [14]. This is in consistent with our study where elevated levels of IL-6 with Mean±SD value of 13.14±3.15 were seen in diabetic patients associated with CAD. Additionally another study examined into whether hypertension could raise the risk of atherosclerosis by causing pro-inflammatory effects by investigating the relationship between blood pressure with baseline plasma concentrations of IL-6 and found a strong correlation between IL-6 levels (P=0.001) [15]. Previous study reported the positive correlation between plasma IL-6 concentration and age (r = 0.51, 0.28, P = 0.001) [16]. Interleukin-6 could induce the production of alpha 1-antichymotrypsin and beta-amyloid protein precursor, and changes in plasma IL-6 might provide a pathological basis for the susceptibility to illnesses [17]. Similarly our study also found significantly strong correlation between IL-6 and BMI, LDL, Uric acid and Hs-CRP (r = 0.434, 0.625, 0.421 and 0.617) (Figure 3). The increasing levels of hs-CRP (Mean/SD=7.35±0.74) in diabetic CAD patients of our study could be due to atherogenic and thrombogenic vascular potential of hs-CRP, and there is a strong evidence that hs-CRP plays a direct role in atherothrombosis aetiology [18]. In vitro, hs-CRP was discovered to be a powerful activator of tissue factor synthesis by macrophages. Because tissue factor is the primary originator of coagulation and atherosclerosis in vivo, its local concentration in the artery wall is linked to coronary atherothrombotic events. As a result, ability of hs-CRP's to stimulate tissue factor production reveals a probable causal relationship between elevated hs-CRP levels and coronary events [19]. Similarly a conducted pilot study on hs-CRP and oxidative stress in young CAD patients in India, and found that increased hs-CRP, combined with dyslipidemia and oxidative stress, improved the predictability of premature CAD [20].

In our study, serum uric acid levels were found to be substantially higher in patients to cause release of free radicals, which have been linked to inflammatory cell adhesion molecule expression, activation, and adhesion to damaged endothelium result of this, which raises the risk of cardiovascular disease [21]. This mechanism is supported by a study conducted by in 39 male patients with chronic heart failure and 16 healthy controls, in which they measured circulating uric acid and inflammation markers and discovered a link between elevated UA levels and chronic inflammation in chronic heart failure [22]. Additionally another study found to an increase in plasma UA concentration is connected with an elevated level of C-reactive protein, which is a significant biomarker of myocardial infarction, stroke, and vascular mortality [23]. Our results with elevated levels of inflammatory cytokines (IL-6 and hs-CRP) delineate that it might play a more crucial role in pro-inflammatory regulation mechanisms. Persistent hyperglycemia contributes to the creation of advanced glycation end products that are known to play a significant role in the development of chronic inflammation [24]. Also, insulin resistance in T2DM patients with increased BMI is linked to increased inflammatory cytokines levels and is considered as a significant pathogenic process in the progress of hypertension [25]. In our study, the increase in BMI might be due to increased circulating IL-6 levels which in turn, low grade inflammation could be a potent inducer in T2DM. A cross sectional study by found higher levels of IL-6 and CRP with increase in BMI, SBP, DBP and other clinical risk factors (P=0.0001) [26]. Similarly other study also found significant correlation between waist - hip ratio and hs-CRP, IL-6 (r=0.294, 0.437p= 0.022 and 0.003) [27]. Similarly, in our study, as IL-6 concentration increased hs-CRP also increased and thus the correlation between IL-6 and Hs-CRP was found statistically significant (r=0.786, p=0.0001).

## Conclusions:

Data shows that the raised levels of blood pressure and diabetes may be provoke for inflammatory cytokines IL-6, hs-CRP, and uric acid which are promising potential biomarkers for detecting CAD in individuals.
